# Embryonic and larval development of the endangered Chiapas killifish *Tlaloc hildebrandi* (Cyprinodontiformes: Profundulidae)

**DOI:** 10.1111/jfb.70632

**Published:** 2026-09-04

**Authors:** Rubén Alonso Contreras‐Tapia, Gabriela Garza‐Mouriño, María Elena Castellanos‐Páez, Marcela Ivonne Benítez‐Díaz Mirón, Jairo Arroyave, Alfonso Ángel González‐Díaz, Wilfredo A. Matamoros

**Affiliations:** ^1^ Laboratorio de Rotiferología y Biología Molecular de Plancton, Departamento El Hombre y su Ambiente Universidad Autónoma Metropolitana, Unidad Xochimilco (UAM‐X) Ciudad de México México; ^2^ Instituto de Biología, Universidad Nacional Autónoma de México (UNAM) Ciudad de México México; ^3^ Colección de Peces, Departamento de Conservación de la Biodiversidad El Colegio de la Frontera Sur San Cristóbal de Las Casas Chiapas México; ^4^ Laboratorio de Diversidad Acuática y Biogeografía, Instituto de Ciencias Biológicas Universidad de Ciencias y Artes de Chiapas (UNICACH) Tuxtla Gutiérrez Chiapas México; ^5^ Instituto Hondureño de Investigación en Ciencias Biológicas y Ambientales de Honduras (IBIOAH), Facultad de Ciencias Universidad Nacional Autónoma de Honduras (UNAH), Campus Cortés San Pedro Sula Honduras; ^6^ Collections Associate, Field Museum of Natural History Chicago Illinois USA

**Keywords:** conservation, Cyprinodontiformes, larval morphology, neotropical, ontogeny, profundulids

## Abstract

The early ontogeny of endemic freshwater fishes provides essential baseline biological information for conservation and management, particularly in species with restricted distributions and high vulnerability to habitat degradation, environmental change and population decline. This study presents the first detailed description of embryonic and larval development of *Tlaloc hildebrandi*, an endangered (EN) killifish endemic to Chiapas, Mexico. Ontogenetic development was documented under controlled conditions from fertilisation to the completion of the larval period and the establishment of juvenile morphology at 35 days post‐hatching. Embryogenesis proceeded through a typical teleost sequence, including cleavage, blastula, gastrulation, somitogenesis and organogenesis, and was completed in approximately 12 days (300 accumulated thermal units, ATU) at 25°C. Eggs were relatively large (2.33 ± 0.03 mm) and demersal, with adhesive filaments concentrated near the micropyle. Larvae hatched at a comparatively large size (7.20 ± 0.04 mm total length) and exhibited advanced morphological features, including functional sensory systems and early feeding capability. A mixed‐feeding period persisted for approximately 5 days, after which larvae transitioned to exclusive exogenous feeding. Post‐hatching development involved progressive differentiation of cranial structures, fins, lateral line system and olfactory organs, culminating in the establishment of juvenile morphology by 35 days post‐hatching. The developmental trajectory of *T. hildebrandi* reflects a non‐annual life history strategy characterised by direct development, absence of diapause and high maternal investment per offspring. Large egg size, extended yolk utilisation and early feeding behaviour likely enhance larval survival in stable, high‐altitude freshwater environments. This study contributes to the knowledge of developmental biology in fishes of the Middle American family Profundulidae and provides a baseline of information for future comparative studies and conservation programs, including captive propagation, larval rearing and population monitoring.

## 1 INTRODUCTION

The study of embryonic and larval development constitutes a fundamental component of conservation biology for endangered and endemic freshwater fishes, as early life‐history stages are often the most sensitive and vulnerable phases of the life cycle (Esmaeili et al., 2025; Weis & Weis, 2018). For short‐range endemic species with restricted distributions, developmental information frequently represents the primary baseline for understanding reproductive biology, survival and responses to environmental conditions under both natural and captive settings (Esmaeili et al., 2025). Detailed descriptions of early ontogeny are therefore essential for the design of effective conservation strategies, including captive breeding and assisted propagation programs, which are increasingly necessary to prevent local or anthropogenic extirpations. Knowledge of embryogenesis and larval development directly supports conservation management by enabling the establishment of ex situ safety stocks and informing translocation and reintroduction efforts. Successful recruitment depends on suitable physicochemical and habitat conditions during early development, including temperature, dissolved oxygen, water chemistry, current velocity, water depth and substrate characteristics (Brown et al., 2011; Kapakos et al., 2024; Karageorgou et al., 2025). In addition, embryos and larvae function as sensitive biological indicators of freshwater ecosystem health, as exposure to contaminants during development can result in morphological abnormalities or functional impairments that may not be evident in adults (Schnitzler et al., 2017; Weis & Weis, 2018). Developmental data also contribute to population assessment and taxonomy, since larval stages often provide reliable diagnostic characters for species identification and allow for the evaluation of reproductive success in natural populations. Furthermore, understanding the timing of yolk‐sac absorption and the transition to exogenous feeding is critical, as this period represents a major survival bottleneck; optimising abiotic conditions to achieve adequate size at hatch can significantly enhance larval survival and recruitment success (Hamre et al., 2013; Houde, 1997; Yúfera & Darias, 2007).

The Chiapas killifish, *Tlaloc hildebrandi* (Miller, 1950), locally known as “popoyote”, is a small freshwater fish of notable biological and conservation significance due to its microendemic distribution and Endangered (EN) conservation status (López‐Villa & González‐Díaz, 2025; Soria‐Barreto et al., 2023). This species belongs to the family Profundulidae, which is one of the few fish families endemic to tropical Middle America. Recent phylogenetic and biogeographic analyses indicate that Profundulidae is the sister lineage of Goodeidae, with divergence estimated at approximately 27 Ma during the Miocene, reflecting a moderately long evolutionary history within Cyprinodontiformes (Hernández‐Ávila et al., 2024). Biologically, *T. hildebrandi* is distinguished by several uncommon anatomical and reproductive traits, including the absence of a stomach and pyloric caeca, the presence of an intestinal valve and a distinctive coronoid cartilage in the jaw apparatus (Domínguez‐Cisneros et al., 2023). Spawning occurs primarily during the dry season (January–June), and females reach first sexual maturity at approximately 41–60 mm total length, producing over 1000 oocytes on average (Cruz‐Maza, 2018; Velázquez‐Velázquez & Domínguez‐Cisneros, 2025).

Ecologically, *T. hildebrandi* plays a key role in high‐altitude freshwater ecosystems of southern Mexico, where it is often the only native fish species present (Beltrán‐López et al., 2021; Piller et al., 2024). The species is a carnivorous‐insectivorous predator, feeding primarily on aquatic invertebrates such as chironomid larvae, ostracods and amphipods, and thus contributes to local trophic dynamics (Velázquez‐Velázquez & Domínguez‐Cisneros, 2025). It occupies both lotic and lentic environments, including alpine wetlands, mountain streams and rivers characterised by rocky substrates and dense aquatic vegetation that provide shelter and breeding sites. Despite its ecological importance, *T. hildebrandi* is threatened and is currently classified as EN by the IUCN Red List and protected under Mexican legislation (NOM‐059‐SEMARNAT‐2010) (Schmitter‐Soto & Vega‐Cendejas, 2019). Additional pressure from invasive species, such as *Micropterus salmoides* and *Cyprinus carpio*, and recently the red swamp crayfish, *Procambarus clarkii*, has further contributed to local extirpations (López‐Villa & González‐Díaz, 2025). Although conservation measures such as Ramsar site designation and protected municipal wetlands have been implemented, the long‐term persistence of *T. hildebrandi* depends on integrated strategies that include habitat restoration and effective ex situ conservation programs (Schmitter‐Soto & Vega‐Cendejas, 2019).

Despite growing interest in the biology and conservation of the genus *Tlaloc*, current knowledge of early development remains limited. Current studies have focused primarily on reproductive parameters and general life‐history traits, while detailed descriptions of embryogenesis and larval development are largely absent. Available information indicates that *Tlaloc* species exhibit an opportunistic reproductive strategy, characterised by partial spawning and rapid maturation. Comparative analyses among the three Mexican species revealed significant interspecific variation in oocyte size, with *T. labialis* producing the largest oocytes, followed by *T. candalarius*, and *T. hildebrandi* exhibiting the smallest mean oocyte diameter (Anzueto‐Calvo et al., 2024). Field observations further suggest prolonged reproductive activity, as evidenced by the presence of very small individuals throughout the year, with a recruitment peak during the dry season (Cruz‐Maza, 2018). Early life stages are also known to be particularly sensitive to environmental stressors, including parasite transmission from invasive exotic species, underscoring the vulnerability of larvae and juveniles in natural populations (Beltrán‐López et al., 2021).

In contrast to the growing body of information on reproductive output and population structure, critical gaps persist regarding the ontogenetic development of *Tlaloc* species. No detailed descriptions currently exist for the internal stages of embryogenesis, including cleavage patterns, gastrulation, or organogenesis. Likewise, larval morphology and developmental timing, such as yolk‐sac absorption, finfold differentiation and the transition from larval to juvenile stages, remain undocumented. These gaps limit the ability to evaluate larval survival, assess population resilience and compare developmental stages among closely related and morphologically similar species. Therefore, the objective of this study is to document and characterise the embryonic and larval development of *Tlaloc hildebrandi*, from fertilisation through the emergence of juvenile morphological traits. By providing a detailed morphological and temporal description of early development, this work aimed to establish a foundational framework for future developmental, ecological and conservation‐oriented research and to support both in situ and ex situ management strategies for this endangered endemic species.

## 2 MATERIALS AND METHODS

### 2.1 Husbandry of *Tlaloc hildebrandi*

Adult individuals of *Tlaloc hildebrandi* were obtained from a breeding stock established in 2019 and maintained at the Colección de Peces, Departamento de Conservación de la Biodiversidad, El Colegio de la Frontera Sur (ECOSUR), San Cristóbal de Las Casas, Mexico. The breeding stock originated from wild‐caught juveniles and was maintained under captive conditions before the initiation of the present developmental study. Fish were maintained in captivity under controlled conditions for breeding and developmental observations. Adults (*n* = 12) were maintained communally under semi‐natural breeding conditions in indoor aquaria. Sexes were distinguished based on external dimorphism and reproductive behaviour (Figure 1). Parentage of individual eggs was not determined.

**FIGURE 1:**
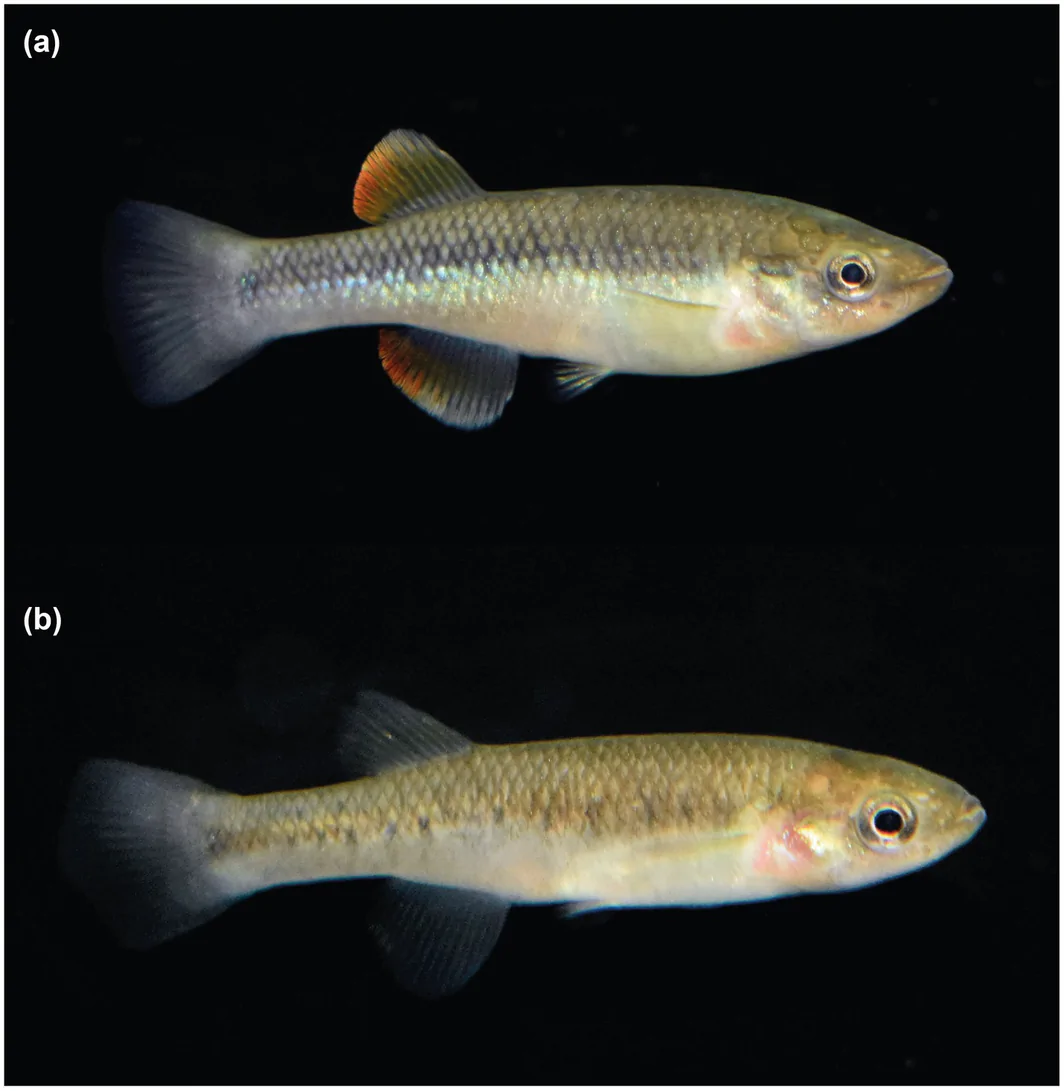
Sexual dimorphism in *Tlaloc hildebrandi*. (a) Adult male showing a modified anal fin with bony spinules and distinct orange pigmentation along the distal margins of the dorsal and anal fins. (b) Adult female lacking marginal pigmentation; the anal fin is elongated.

Fish were initially maintained for approximately 6 months in 240‐L indoor aquaria and subsequently transferred to outdoor tanks, where they were kept for 1 year before egg collection began. The main outdoor tank had a capacity of 500 L and housed all breeding adults. Additional juveniles were maintained in a separate 300‐L outdoor tank. Outdoor tanks were maintained on a rooftop in Mexico City and were exposed to natural photoperiod, partial shade and direct rain input. Water consisted of dechlorinated tap water. Water quality was maintained by weekly 10%–15% water exchanges using conditioned water that had been continuously aerated before use. Reproductive activity was seasonal and occurred primarily between February and May, with a secondary period from August to September, when water exceeded approximately 18°C. Water temperature was not actively controlled. pH ranged from 7.8 to 8.0. Dissolved oxygen was not quantitatively measured, although continuous aeration was provided. No filtration system or artificial substrate was used. Adult fish were fed twice daily with a commercial omnivorous diet (El Pedregal; 42% protein content). Preliminary breeding attempts were conducted between 2022 and 2023 to optimise spawning conditions. Systematic egg collection and developmental observations were conducted during 2024.

### 2.2 Spawning, egg collection and incubation

Spawning occurred on submerged egg traps made solely of the aquatic plant *Ceratophyllum demersum*. The plants were anchored within an enclosed refuge located at the centre of the outdoor tank. No other plants or substrates were present. Egg collection was initially assessed using both floating and submerged mops; comparative trials showed that plant‐based egg traps were more effective and were therefore used throughout the study. Egg deposition was monitored daily, and eggs were collected within minutes of detection. Fertilisation was external; females deposited eggs, which were subsequently fertilised by males. Developmental time was standardised from the first detection of eggs in the spawning trap. Eggs were deposited seasonally, with an average of 25–50 eggs collected per day during peak reproductive periods. Filial cannibalism was observed in outdoor tanks, but decreased under green‐water conditions associated with microalgal growth; shade netting was used to regulate light intensity. Immediately after collection, eggs were transferred to the laboratory, where all embryonic development was conducted under controlled conditions. Each egg was individually placed into a 24‐well cell culture plate (Sarstedt, Germany) containing 2.5 mL of EPA medium (USEPA, 1985). Eggs were incubated at a constant temperature of 25°C under artificial light with a 12:12‐h light: dark photoperiod. The incubation medium was renewed daily to maintain water quality and adequate oxygen availability. A total of 250 embryos were monitored. Eggs were collected across multiple spawning events and from multiple females; each embryo was treated as an independent developmental unit. Embryos were observed every 30 min during the first 6 h post‐fertilisation, and subsequently every 6 h until hatching. Mortality was recorded daily. Hatching success was calculated as the percentage of successfully hatched embryos relative to the total number incubated and was 90%.

### 2.3 Larval rearing and feeding

Newly hatched larvae were grouped into cohorts according to hatching date and transferred to polypropylene containers (120 mL capacity, 100 mL medium), with a maximum density of 10 larvae per container (density = 1 larva/10 mL). When fewer larvae hatched on a given day, density was proportionally reduced. Larvae originated from multiple spawning events and were grouped exclusively by age to standardise developmental comparison. Larvae were maintained in EPA medium with daily 50% medium renewal supplemented with *Chlorella vulgaris* at a concentration of 0.25 × 10^6^ cells mL^−1^. Feeding began on the first day post‐hatching (dph). The initial diet consisted of the rotifer *Brachionus angularis*, followed by the cladoceran *Moina macrocopa*. Live prey was cultured in the laboratory and renewed daily in conjunction with water changes. Details on the culture and maintenance of microalgae and zooplankton used as live food are provided in Contreras‐Tapia et al. (2024). A total of 125 larvae were monitored throughout development. Larval survival to the end of the larval period was 85%. The end of the larval period was defined as the stage at which all fins were fully differentiated (ossified fin rays and total resorption of the primordial finfold).

### 2.4 Observation, imaging and morphometrics

Embryonic and larval development was documented using a stereomicroscope equipped with a high‐resolution digital camera (Infinity 8, AccuScope). All images and morphometric measurements were obtained using Image‐Pro v.11 (Media Cybernetics, USA). All measurements were calibrated with digital imaging procedures. Morphometric variables included egg diameter, total length (TL) and standard length (SL). Egg diameter was measured from 60 randomly selected eggs. TL and SL were measured from 15 individuals every 3 days. Measurements were obtained from live specimens using digital images.

Developmental stages were defined based on external morphological criteria established in this study, including cleavage pattern, somite formation, pigmentation, yolk sac absorption and finfold differentiation. Hatching was defined as the complete emergence of the embryo from the chorion. Ontogenetic growth was characterised using growth curves based on mean TL across developmental time (days).

Scanning electron microscopy (SEM) was used to examine surface morphology at selected developmental stages, from hatching to the end of the larval period. Specimens were sampled at 0, 1, 3, 9, 14, 17, 20, 25, 30 and 35 dph, with six individuals examined per stage. Fixation and preparation for SEM followed the same protocol described by Contreras‐Tapia et al. (2025). SEM imaging was conducted using a Hitachi Stereoscan SU1510 SEM at 10 kV at the Laboratorio de Microscopía y Fotografía de la Biodiversidad 1 (LANABIO) of the Instituto de Biología, Universidad Nacional Autónoma de México.

### 2.5 Ethics statement

All procedures were performed in accordance with strict standards for animal care and research established by the Mexican government (NOM‐062‐ZOO‐1999) and the International Council for Laboratory Animal Science (ICLAS). Collection and handling of *Tlaloc hildebrandi* were authorised by the Mexican environmental authorities under scientific collection permit No. SBRA/DGVS/03497/25 (SEMARNAT) and the corresponding scientific fishing permit No. PPF/DGOPA‐076/21 issued to El Colegio de la Frontera Sur (ECOSUR).

## 3 RESULTS

### 3.1 Reproductive context

*Tlaloc hildebrandi* exhibited a seasonal, iteroparous reproductive strategy under captive conditions. Spawning occurred primarily when water temperatures exceeded approximately 18°C and was restricted to distinct seasonal windows, with no egg deposition observed during the rainy season. Females produced multiple egg batches during the reproductive period, depositing approximately 12–15 eggs per day, indicating fractional spawning. Fertilisation was external. Courtship behaviour included chasing, nudging and display movements, after which females deposited adhesive eggs on submerged vegetation within the spawning trap. Males frequently entered the spawning site during oviposition, suggesting immediate fertilisation; however, parentage of individual clutches was not determined. Although males displayed increased territoriality during the breeding season, multiple breeding pairs utilised the same spawning substrate. Eggs were demersal and adhesive, attaching to vegetation by mucous filaments concentrated near the micropyle. Eggs were deposited in small clusters. No parental care was observed after oviposition. Females reached apparent sexual maturity at approximately 60 mm total length, corresponding to an age of roughly 11 months under captive conditions.

### 3.2 Embryonic development

#### 3.2.1 Stage 1. Fertilised egg (0 hpf )

Eggs were spherical, transparent and measured 2.33 ± 0.03 mm in diameter. The chorion was smooth under light microscopy and lacked surface ornamentation. Adhesive filaments were concentrated exclusively around the micropyle region and facilitated attachment to vegetation at oviposition (Figure 2a). These filaments persisted after fertilisation and remained functional, allowing eggs to adhere to each other or to residual substrate material during handling. A single, centrally located micropyle was present at the animal pole and appeared as a distinct, funnel‐shaped depression (Figure 2b). Within 30–45 min post‐fertilisation, the chorion hardened and a conspicuous perivitelline space formed uniformly between the chorion and the vitelline membrane. The perivitelline space expanded symmetrically around the yolk mass (Figure 3a). Cytoplasmic streaming toward the animal pole occurred during the first 1.5–2 h post‐fertilisation (hpf), resulting in the formation of a sharply delimited, cap‐like blastodisc. The blastodisc was slightly elevated above the yolk surface and clearly demarcated from the vegetal region, establishing a visible animal–vegetal axis. No cleavage furrows were present during this stage. The yolk contained one or two large central oil droplets accompanied by two to three smaller droplets, which were clustered and non‐motile. Stage 1 extended from fertilisation until the appearance of the first meroblastic cleavage furrow, which occurred at approximately 2.5–3 hpf.

**FIGURE 2:**
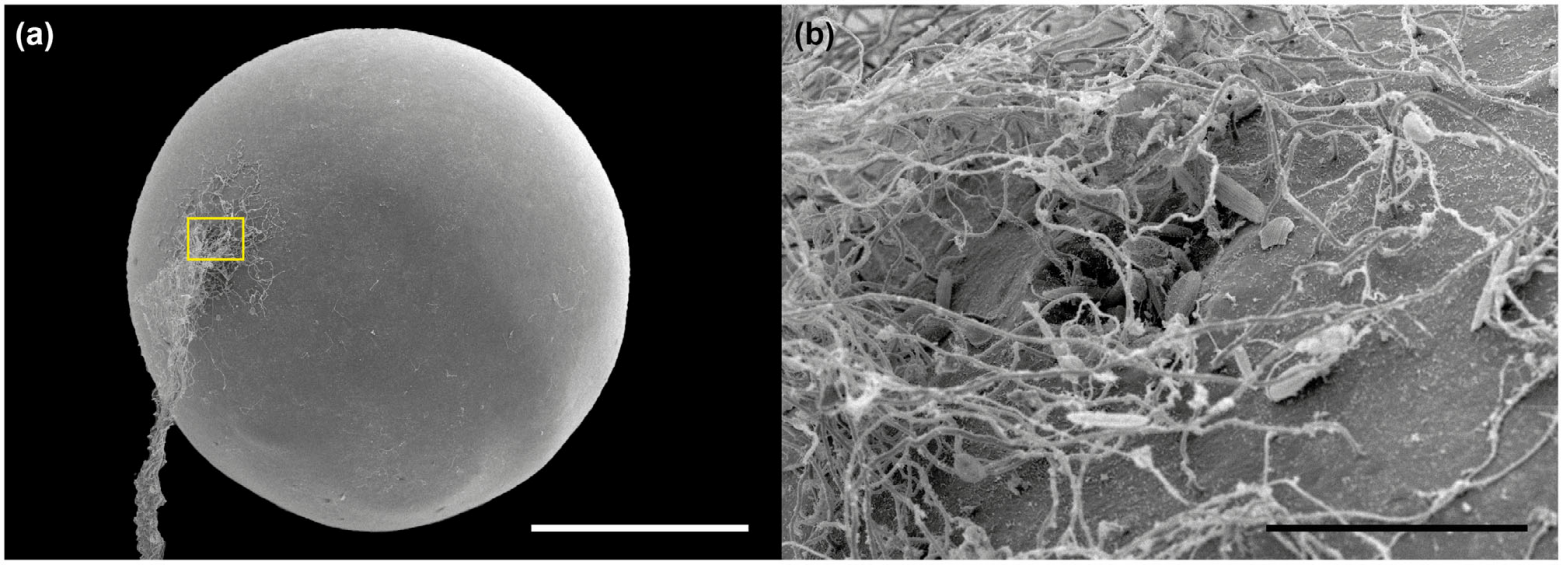
Egg surface structures of *Tlaloc hildebrandi* observed by scanning electron microscopy (SEM). (a) Entire egg showing the micropyle region and adhesive filaments; (b) detailed view of the egg micropyle. Scale bars: *a* = 1 mm; *b* = 100 μm.

**FIGURE 3:**
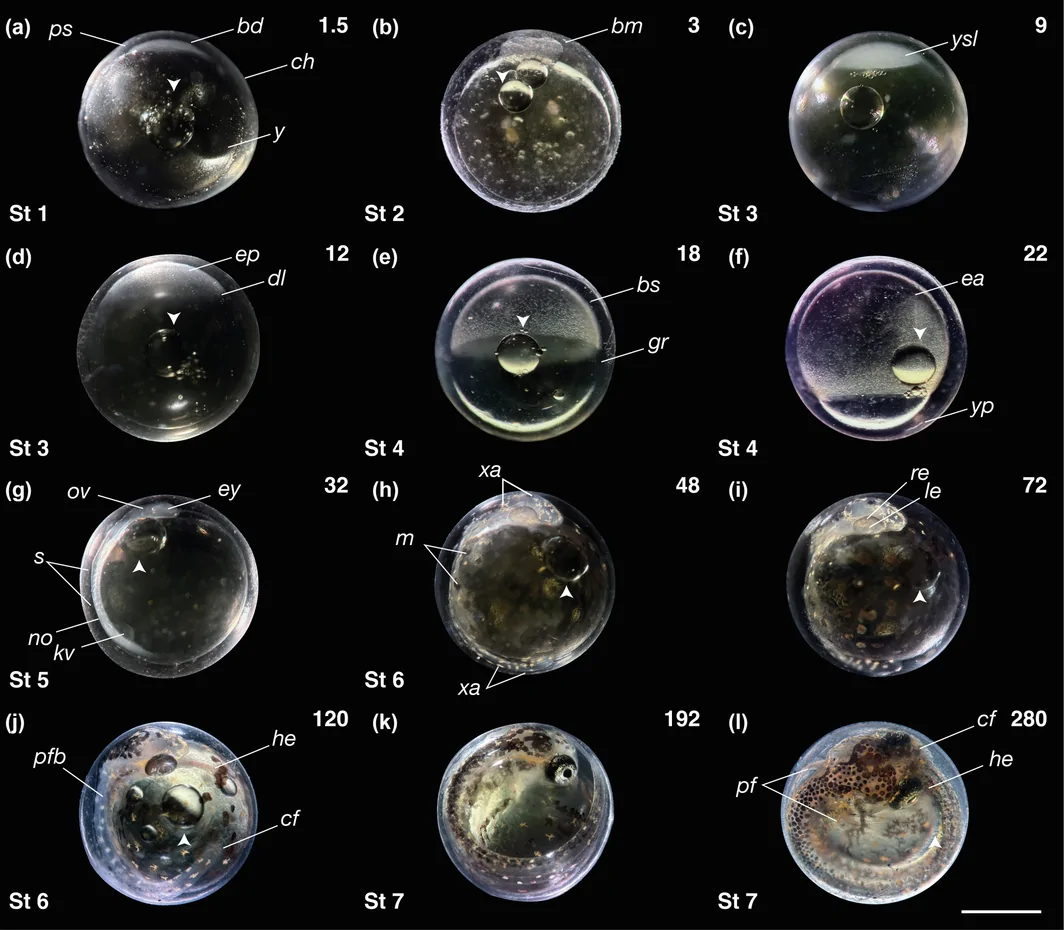
Micrographs of some of the key developmental stages during embryonic development of *Tlaloc hildebrandi*. Stage numbers are indicated at the bottom left, and time in hours post‐fertilisation (hpf) at the upper right. Arrowheads indicate an oil droplet. bd = blastodisc; bm = blastomere; bs = blastoderm; ch = chorion; cf. = caudal fin; dl = dorsal lip; ea. = embryonic axis; ep = epiboly; ey = eye; gr = germ ring; he = heart; kv = Kupffer's vesicle; le = lens; m = melanophores; no = notochord; pf = pectoral fin; pfb = pectoral fin bud; ps = perivitelline space; re = retina; s = somites; ov = otic vesicle; xa = xantophores; y = yolk; yp = yolk plug; ysl = yolk syncytial layer. Scale bar = 1 mm.

#### 3.2.2 Stage 2. Cleavage (3–8 hpf )

Cleavage was meroblastic and restricted to the blastodisc at the animal pole. The first cleavage furrow appeared at approximately 2.5 hpf and was meridional, bisecting the blastodisc into two equal blastomeres. The second cleavage, occurring between 2:50 and 3:20 hpf, was perpendicular to the first, producing a 4‐cell stage. Subsequent divisions followed the typical discoidal teleost pattern, generating 8‐cell (3:20–3:45 hpf), 16‐cell (3:45–4:30 hpf), 32‐cell (4:30–5:30 hpf) and 64‐cell stages (5:30 hpf; Figure 3b). Cleavage furrows were complete across the blastodisc but did not extend into the underlying yolk mass. Early blastomeres were rounded, forming a compact, solid cellular mass. By the 32‐cell stage, multiple tiers of blastomeres were evident. Cleavage appeared largely synchronous through the early divisions; however, synchrony became less apparent after the 32‐cell stage. During late cleavage, blastomeres in the outer layer began to flatten and expand laterally, establishing close intercellular contact and producing a smoother blastoderm surface. No spreading of the blastoderm over the yolk was observed during this stage. Oil droplets remained clustered centrally within the yolk and showed no evident migration during cleavage. Stage 2 extended to approximately 8 hpf and was characterised by successive meroblastic divisions, culminating in the onset of blastoderm flattening, which marked the transition to the blastula stage.

#### 3.2.3 Stage 3. Blastula (8–15 hpf )

At approximately 8 hpf, the blastoderm began to flatten progressively against the yolk mass, transitioning from the compact, multilayered cleavage mass into a smooth, discoidal structure clearly demarcated from the underlying yolk. Blastomere thickness decreased as cells expanded laterally, producing a distinct blastoderm with a sharply defined margin. By 12 hpf, an inner segmentation cavity became visible within the blastoderm. This cavity appeared centrally located beneath the superficial cell layers and expanded progressively during the stage. The blastoderm remained confined to the animal pole and did not yet spread over the yolk surface. At the blastoderm margin, a thickened cellular ring was evident, consistent with the formation of a yolk syncytial layer (YSL, Figure 3c). Marginal blastomeres appeared to fuse with the yolk cytoplasmic layer, producing a distinct boundary between blastoderm and yolk. Concurrently, oil droplets gradually migrated away from the central yolk region toward the marginal zone adjacent to the developing YSL. This relocation was progressive and became clearly evident by 12 hpf. Stage 3 extended until the onset of epiboly, defined by the initial vegetal expansion of the blastoderm over the yolk surface.

#### 3.2.4 Stage 4. Gastrula and epiboly (15–24 hpf )

Gastrulation commenced at approximately 12 hpf with the initiation of epiboly, marked by the vegetal expansion of the blastoderm over the yolk surface. At 25% epiboly (14–16 hpf), a distinct and continuous germ ring formed circumferentially at the blastoderm margin. The germ ring appeared as a well‐defined, thickened band that progressively intensified as epiboly advanced. Concurrently, an embryonic shield became visible on the dorsal side at approximately 25% epiboly. The shield appeared as a localised, elongated thickening within the germ ring and established the embryonic axis. Dorsoventral asymmetry was evident from this stage onward, and axis orientation remained fixed throughout subsequent development. Epiboly progressed steadily, reaching 50% coverage between 16 and 20 hpf (Figure 3e) and 75% coverage between 20 and 24 hpf. During mid‐gastrulation, deep blastomeres displayed large, amoeboid morphologies consistent with active cell migration. The germ ring remained clearly defined through 75% epiboly. At approximately 75% epiboly, a prominent, circular yolk plug became visible at the vegetal pole (Figure 3f). The yolk plug decreased gradually as epiboly continued. Complete epiboly (100% coverage) and closure of the yolk plug occurred between 24 and 26 hpf. Stage 4 was defined by progressive epibolic movement of the blastoderm and concluded upon complete enclosure of the yolk at 100% epiboly.

#### 3.2.5 Stage 5. Early‐somite (neurula) stage (24–48 hpf )

The early‐somite stage began between 24 and 26 hpf with the appearance of the first paired somites along the embryonic axis. Somitogenesis proceeded bilaterally and symmetrically, and by the end of this stage, embryos exhibited approximately 10–15 somite pairs. A solid neural keel became visible along the dorsal midline at approximately 30 hpf, marking active neurulation. The embryonic axis elongated progressively, and by ~32 hpf, distinct head and tail bud regions were clearly recognisable. At this time, the embryo began to elevate slightly from the yolk surface. Kupffer's vesicle was prominent in the posterior region around 32 hpf. The vesicle appeared as a distinct, rounded structure at the tail bud and was most evident during early somitogenesis. Optic vesicles became visible at approximately 36 hpf as paired, shallow lateral bulges emerging from the forebrain region. Otic placodes were also evident bilaterally. Stage 5 concluded at approximately 48 hpf, defined by the attainment of 10–15 somite pairs and the onset of cardiac activity, marking the transition to active organogenesis.

#### 3.2.6 Stage 6. Mid‐embryonic stage (organogenesis) (48–192 hpf )

Stage 6 began at approximately 48 hpf with the onset of cardiac activity. The heart differentiated into a functional tubular structure exhibiting rhythmic contractions, and visible blood circulation through the developing vitelline vasculature was established between 50 and 52 hpf. Somitogenesis continued during early organogenesis, and regional differentiation of the central nervous system became apparent, with distinct forebrain, midbrain and hindbrain domains. Optic vesicles progressively invaginated to form optic cups, and lens formation became evident. Retinal pigmentation began around 4 dpf. Otic vesicles were clearly visible by ~72 hpf and contained discernible otoliths. Pigmentation initiated at approximately 48 hpf along the head and trunk. Early melanophores were dendritic in morphology, and xanthophores appeared concurrently. Pigment distribution increased progressively from anterior to posterior regions during development. Spontaneous muscular contractions were first observed around 60 hpf, particularly in the tail region. By approximately 72 hpf, the tail extended beyond the yolk surface and detached from it, and the embryo encircled roughly half of the yolk sphere. Body elongation continued steadily thereafter. Pectoral fin buds became visible at approximately 120 hpf (5 dpf). Opercular movements began around 168 hpf, coinciding with increased embryonic activity. As development progressed toward 192 hpf, the embryo exhibited complete encirclement of the yolk and increased pre‐hatching movements. Stage 6 concluded when the embryo fully encircled the yolk mass and displayed pronounced pre‐hatching activity, marking the transition to the pre‐hatching stage.

#### 3.2.7 Stage 7. Pre‐hatching stage (192–288 hpf )

The pre‐hatching stage was characterised by complete embryonic morphogenesis and pronounced behavioural activity within the chorion. By approximately 8 dpf, the eyes were fully pigmented, exhibiting dark retinas surrounded by a distinctive reflective gold ring encircling the iris. Jaw development progressed visibly from the lower to the upper jaw, and by 8 dpf, the oral cavity was open. Pectoral‐fin rays were evident by 9 dpf, and caudal‐fin ray formation was clearly established. Circulation within the caudal fin was visible by approximately 10 dpf, reflecting maturation of the peripheral vasculature. The gallbladder was distinct, indicating advanced differentiation of the digestive system. The embryo encircled the yolk almost completely, and the yolk mass was markedly reduced compared to earlier stages. The tail extended well beyond the head, following the curvature of the chorion. Opercular movements and active buccal pumping were observed, and overall embryonic activity increased substantially. Frequent body rotations and vigorous tail movements were evident, particularly after 10 dpf. Stage 7 concluded at approximately 12 dpf (288 hpf), when vigorous tail lashing resulted in rupture of the chorion and initiation of hatching.

#### 3.2.8 Stage 8. Hatching (288 hpf; 12 dpf )

Hatching occurred at approximately 288 hpf (12 dpf). Shortly before emergence, embryos exhibited vigorous body rotations and repeated tail thrashing within the chorion. Chorion rupture occurred due to mechanical stress generated by forceful tail movements, resulting in a tear in the egg envelope. Hatching was consistently tail‐first. A swim bladder was visible several hours before hatching. At the time of emergence, the yolk sac was approximately half absorbed. The newly hatched larvae retained a continuous finfold extending from the dorsal to ventral margin of the body. The tail was well elongated, and fin rays in the caudal and pectoral fins were already present. Larvae emerged fully pigmented, with darkly pigmented eyes encircled by a reflective gold ring. Opercular movements were active immediately following hatching. Stage 8 was defined by the complete exit of the embryo from the chorion and the transition to free‐living larval life.

#### 3.2.9 Stage 9. Mixed feeding period (0–5 dph)

Newly hatched larvae measured 7.20 ± 0.04 mm total length (TL) and retained an ovoid yolk sac containing visible oil droplets and extensive vascularisation. A continuous larval finfold was present as a thin, transparent membrane extending dorsally and ventrally along the body; dorsally from approximately mid‐body length and ventrally extending anteriorly toward the yolk sac and including the pre‐anal region. Pectoral fins were well developed at hatching, and the caudal fin possessed 12 rays. Immediately after emergence, larvae rested on the bottom for approximately 30 min before initiating intermittent swimming behaviour. Within 30–60 min post‐hatching, active feeding behaviour was observed, indicating functional exogenous feeding despite the persistence of yolk reserves. Although gut contents were not visibly distinguishable immediately after the first feeding, food particles were clearly present within the digestive tract during subsequent days. The digestive tract appeared as a straight tubular structure extending along the body axis and exhibited visible peristaltic movements. The gallbladder was distinct at hatching. The oral cavity was functional at emergence; however, the upper and lower jaws were not fully differentiated, and the lower jaw continued developing during the first days post‐hatching. By 5 dph, the lower jaw was more developed and lower lips were evident. Sensory structures were already evident at hatching. The olfactory pit appeared as an ellipsoidal invagination (50 μm in major axis) densely packed with cilia and deepened progressively during the first days. Several superficial mandibular, supraorbital and supranasal neuromasts were present. Pigmentation increased progressively during this stage. Melanophores and xanthophores expanded in number and distribution across the head and trunk. Larvae reached 8.392 ± 0.08 mm TL by 5 dph. Yolk reserves were fully absorbed by 5 dph, marking the end of mixed endogenous–exogenous nutrition and the transition to exclusive exogenous feeding. Stage 9 was defined by the coexistence of residual yolk reserves and active feeding behaviour and concluded upon complete yolk absorption at 5 dph.

#### 3.2.10 Stage 10. Exogenous feeding (5–35 dph)

Following complete yolk absorption at 5 dph, larvae entered a phase of exclusive exogenous feeding accompanied by progressive morphological differentiation and fin development. During this period, total length increased from 8.392 ± 0.08 mm at 5 dph to 14.265 ± 0.55 mm by 35 dph. By 9 dph, the upper lip was formed, and a fold of skin connecting the dorsal head region to the upper lip became evident. This protrusion increased in size and flexibility in subsequent days. The mouth gradually attained a terminal position. From 17 to 30 dph, the protrusible structure became more developed, and by 35 dph, it was fully formed and extensible. Concurrently, the lips became thicker and more defined. The caudal peduncle became progressively more defined, and the swim bladder appeared elongated. Squamation began at 35 dph, when cycloid scales first became visible. Stage 10 concluded with the attainment of a fully functional feeding apparatus and the onset of squamation, marking the transition toward juvenile morphology.

#### 3.2.11 Stage 11. End of development (35 dph)

By 35 dph, individuals had completed the larval‐to‐juvenile transition. Body proportions were fully established, swimming behaviour was sustained and coordinated, and feeding was entirely exogenous. The body form closely resembled that of adults, although definitive adult pigmentation patterns were not yet fully expressed. Sexual dimorphism was not clearly distinguishable at this stage. Stage 11 was defined by the attainment of juvenile body morphology and functional independence, marking the end of larval development (Figure 4d).

**FIGURE 4:**
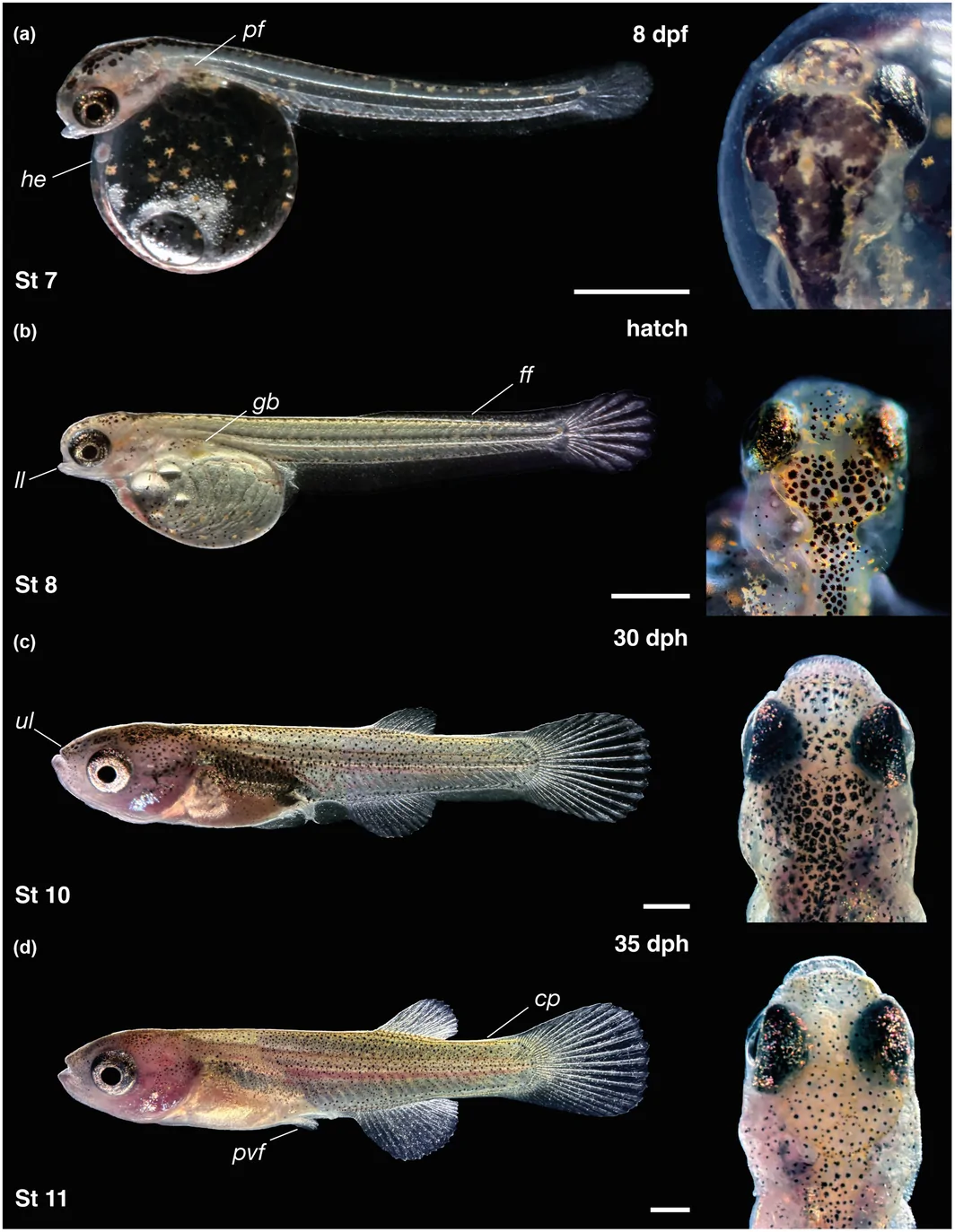
Ontogenetic progression of *Tlaloc hildebrandi* from the pre‐hatching stage to juvenile morphology. (a) Dechorionated embryo at Stage 7; (b) larvae at hatching; (c) larvae at 30 dph; (d) larvae at 35 dph. Stage number is indicated in the lower left corner, and developmental time (hpf or dph) in the upper right corner of each image. cp = caudal peduncle; ff = finfold; gb = gallbladder; ll = lower lip; pf = pectoral fins; pvf = pelvic fins; ul = upper lip. Scale bar = 1 mm.

### 3.3 Head morphogenesis

Head development in *Tlaloc hildebrandi* showed a progressive transition from a rounded larval morphology at hatching to a more elongated and functionally specialised juvenile condition by 35 dph (Figure 5a–f). At hatching (0 dph), the head was short and rounded, with a relatively large, fully formed spherical eye occupying a substantial proportion of the head (Figure 5a). The mouth was open; however, the upper and lower jaws were not yet distinguishable. Branchiostegal membranes were united anteriorly and remained free from the isthmus (Figure 5g). By 3 dph, the head began to elongate, and the lower jaw became distinguishable, marking the onset of jaw differentiation (Figure 5b). Teeth began to emerge at this stage (Figure 5m), and by 6 dph, conical teeth were clearly evident (Figure 5n). During the early larval period (0–5 dph), the head retained a generally rounded profile, although elongation became increasingly evident (Figure 5a–f). By 9 dph, the upper jaw became distinguishable, and both upper and lower lips began to develop (Figure 5c). Teeth were clearly defined at this stage. The mouth was positioned slightly superior during early development but progressively shifted toward a terminal position. Between 9 and 11 dph, the lips became well developed and clearly separated. From 17 to 30 dph, a fold of skin connecting the dorsal head region to the upper lip developed (Figure 5d,e), forming a protrusible structure that increased in size and flexibility. By 35 dph, this structure was well developed and extensible (Figure 5f). Concurrently, the mouth reached its final terminal position, and the lips became thicker and more defined. The relative size of the eye decreased during development as the head grew, although the eye remained spherical throughout all stages. Taste buds were present as early as 3 dph (Figure 5m) and increased progressively in number throughout development (Figure 5o).

**FIGURE 5:**
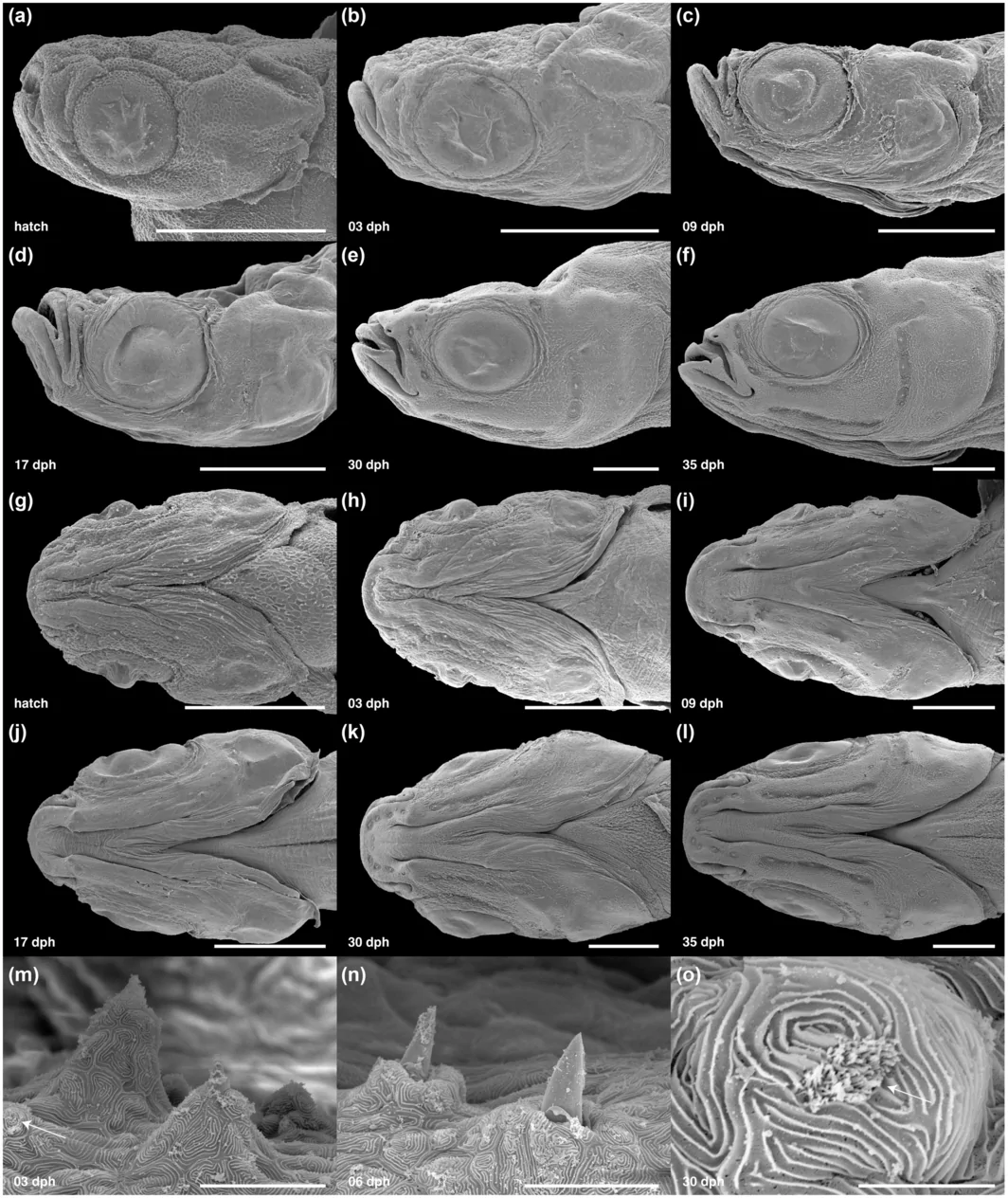
Head morphogenesis of *Tlaloc hildebrandi* from hatching to 35 days post‐hatching (dph). (a–f) Lateral views showing progressive head elongation and oral development: (a) hatching (0 dph); (b) 3 dph; (c) 9 dph; (d) 17 dph; (e) 30 dph; (f) 35 dph. (g–l) Ventral views of the head at corresponding stages: (g) hatching; (h) 3 dph; (i) 9 dph; (j) 17 dph; (k) 30 dph; (l) 35 dph, illustrating changes in jaw structure and branchiostegal region. (m–n) Detail of tooth development: (m) initial tooth emergence at 3 dph; (n) conical teeth at 6 dph; (o) Detail of taste buds at 30 dph. White arrows indicate taste buds. Scale bars: (a–l) = 500 μm; (m, n) = 20 μm; o = 5 μm.

### 3.4 Development of the lateral line system

The lateral line system of *Tlaloc hildebrandi* exhibited a progressive development from a simple cranial arrangement at hatching to a complex pattern of superficial and canal neuromasts by 35 dph (Figure 6). At hatching, the lateral line system was in an early stage of patterning and consisted of a limited number of primary neuromasts restricted to the cranial region. These neuromasts were primarily located in the mandibular and preorbital regions, with no evident neuromasts along the trunk lateral line. Early neuromasts were small and rounded, each consisting of a central cluster of hair cells bearing a kinocilium and multiple stereocilia, surrounded by supporting cells (Figure 6d). During early larval development, the number of neuromasts increased progressively, expanding across the head. By 35 dph, neuromasts were arranged in well‐defined cranial series, including preorbital, suborbital, supraorbital, infraorbital, mandibular, preopercular and opercular regions (Figure 6a–c). This arrangement reflects the establishment of the adult‐like cranial lateral line pattern. A clear differentiation between superficial neuromasts and canal neuromasts became evident during development. Superficial neuromasts remained on the epidermal surface and increased in number throughout the larval period. These structures were generally small and rounded, with their apical surfaces becoming level with the surrounding epithelium as development progressed (Figure 6d,e). In contrast, presumptive canal neuromasts increased in size and adopted an elongated morphology aligned with the future canal axis. These neuromasts underwent a sequence of morphological changes associated with canal formation. Initially positioned on the flat epithelial surface, they gradually became embedded within shallow depressions and subsequently deeper grooves (Figure 6f,g). Canal formation proceeded through progressive enclosure of these grooves, leading to the development of enclosed canals with external pores connecting the canal lumen to the surrounding environment. The development of canal segments appeared to follow a rostro‐caudal progression, with anterior cranial regions differentiating earlier than posterior regions. Canal neuromasts were observed in association with defined cranial structures, including the mandibular and opercular regions (Figure 6f,g).

**FIGURE 6:**
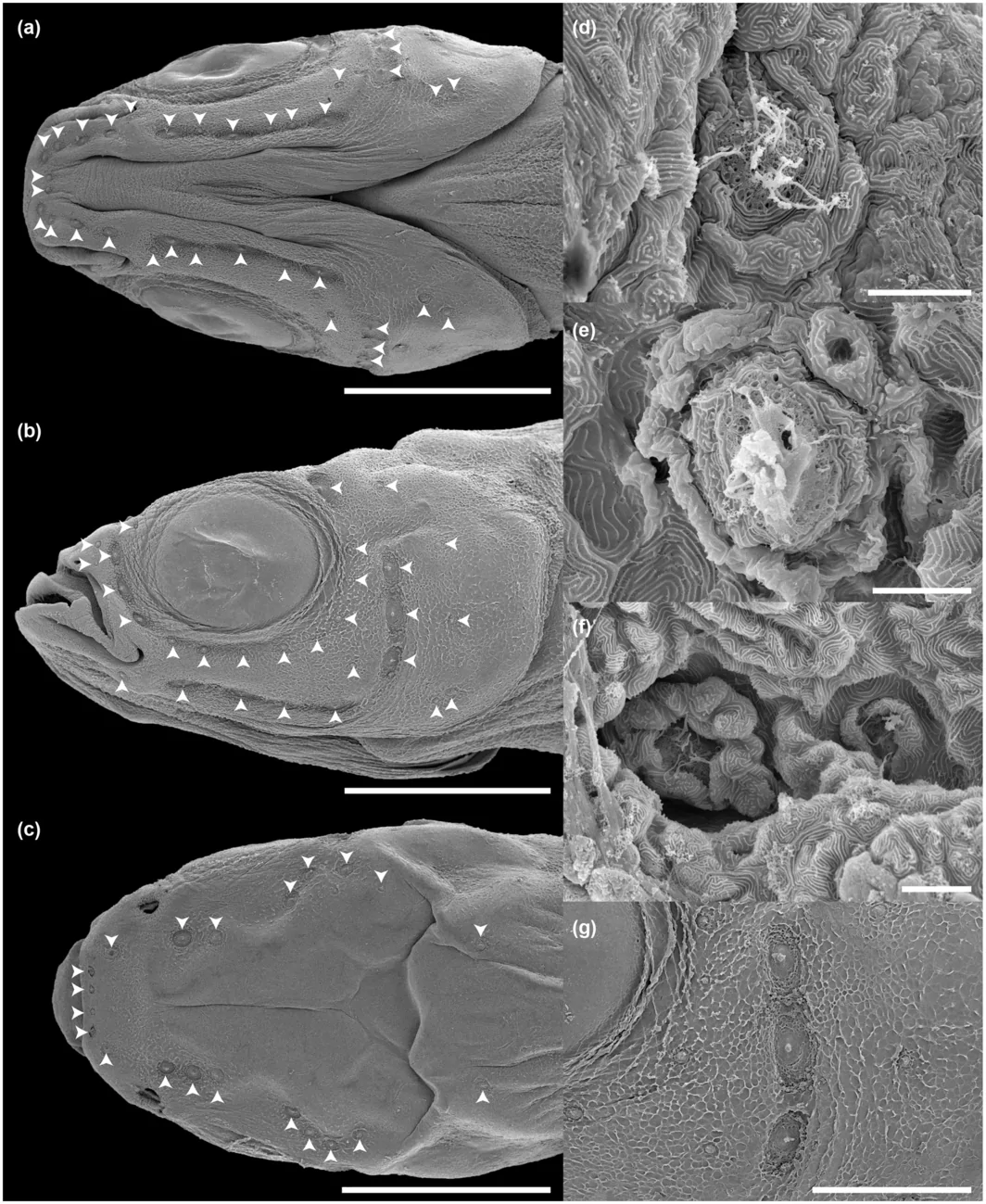
Development of the cranial lateral line system in *Tlaloc hildebrandi*. (a–c) Distribution and arrangement of cranial neuromasts at 35 dph: (a) ventral view; (b) lateral view; (c) dorsal view. Arrowheads indicate the position of neuromasts, including preorbital, suborbital, supraorbital, infraorbital, mandibular, preopercular and opercular series. (d–g) Morphology of neuromasts: (d) superficial neuromast; (e) lateral line neuromast along the trunk; (f) mandibular canal neuromast; (g) opercular canal neuromast. Scale bars: (a–c) = 1 mm; (d–f) = 10 μm; g = 400 μm.

### 3.5 Ontogeny of the olfactory organ

At hatching (0 dph), the olfactory pit is visible as a shallow, ellipsoidal invagination (Figure 7a). The floor of the pit contains individual olfactory knobs, protruding from the surface and bearing long, radiating sensory cilia. The pit is surrounded by indifferent epithelium characterised by a distinct network of microridges. By day 17 dph, the olfactory pit deepens significantly and assumes a drop‐like configuration (Figure 7b). A critical developmental transition occurs around day 20 dph: the original olfactory pit begins to differentiate into the anterior nostril, while a secondary invagination that will become the posterior nostril starts to form. At this stage, the two openings are separated, but the posterior invagination is not yet open to the exterior. By day 35 dph, the olfactory organs reach a definitive state of development (Figure 7c). The anterior incurrent nostril has differentiated into a short tubular structure with projected edges (Figure 7e), while the posterior excurrent nostril remains a much larger, typically elongated opening (Figure 7d).

**FIGURE 7:**
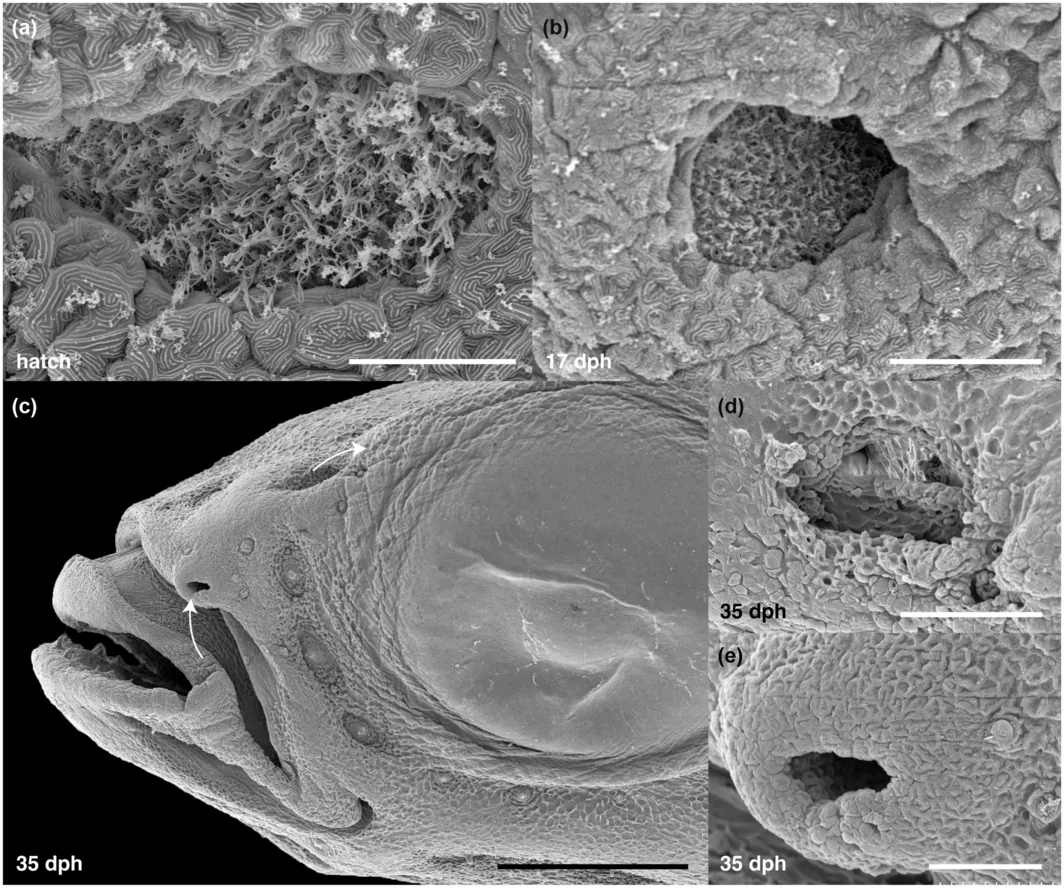
Morphogenesis and ultrastructural development of the olfactory organ in *Tlaloc hildebrandi*. (a) Scanning electron micrograph (SEM) of the olfactory pit at hatching (0 dph), appearing as a shallow ellipsoidal invagination. Note the individual olfactory knobs protruding from the pit floor bearing long radiating sensory cilia, surrounded by an indifferent epithelium characterised by a network of microridges. (b) Olfactory pit at 17 dph showing significant deepening into a gourd‐shaped configuration. (c) Lateral view of the head of *T. hildebrandi* at 35 dph, where the anterior and posterior nostrils are fully differentiated. White arrows indicate the direction of the incurrent and excurrent water currents. (d) High‐magnification view of the posterior (excurrent) nostril. (e) High‐magnification view of the anterior (incurrent) nostril. Scale bars: a, b = 20 μm; c = 400 μm; d = 100 μm; e = 50 μm.

### 3.6 Fin development

Fin development in *Tlaloc hildebrandi* followed a progressive sequence typical of cyprinodontiform fishes, beginning with the differentiation of median fins and concluding with the appearance of the paired pelvic fins and complete resorption of the larval finfold (Figure 8). At hatching, larvae possessed a continuous primordial finfold extending dorsally and ventrally along the body, forming a thin, transparent membrane connecting the dorsal, caudal and ventral regions. The caudal fin already exhibited early differentiation, with 12 identifiable fin rays, and pectoral fins were well developed (Figure 8a). Differentiation of the anal fin began at approximately 13 dph, when the first fin ray primordia became visible (Figure 8b). By 20 dph, dorsal fin differentiation was initiated, with the appearance of fin ray primordia (Figure 8c), while the anal fin was more advanced in development. At this stage, both dorsal and anal fins remained connected to the persistent larval finfold. Concurrent with median fin development, the caudal fin underwent progressive differentiation (Figure 8g–j). Cartilaginous elements of the hypural complex formed at the base of the tail, followed by the appearance of bony fin rays (lepidotrichia) (Figure 8h). The number of caudal fin rays increased steadily during development (Figure 8j). Supporting skeletal elements, including hypurals, parahypurals and epurals, became increasingly defined, providing structural support for the expanding fin rays. As development progressed, the caudal fin gradually acquired its definitive rounded morphology (Figure 8i). Regression of the larval finfold began dorsally (Figure 8d). By 30 dph, the dorsal fin had largely separated from the finfold, although a residual dorsal fold remained continuous with the caudal fin (Figure 8e). Ventrally, the anal fin was well developed but still partially connected to the ventral finfold. Pelvic fin buds appeared at approximately 30 dph, representing the last paired fins to initiate development (Figure 8m). By 35 dph, the primordial finfold was completely resorbed, and all fins were fully differentiated (Figure 8d). Final fin ray counts were: dorsal = 12, anal = 16, caudal = 32, pectoral = 16 and pelvic = 8. At this stage, the caudal peduncle was well defined (Figure 8f), completing the transition to the juvenile fin configuration.

**FIGURE 8:**
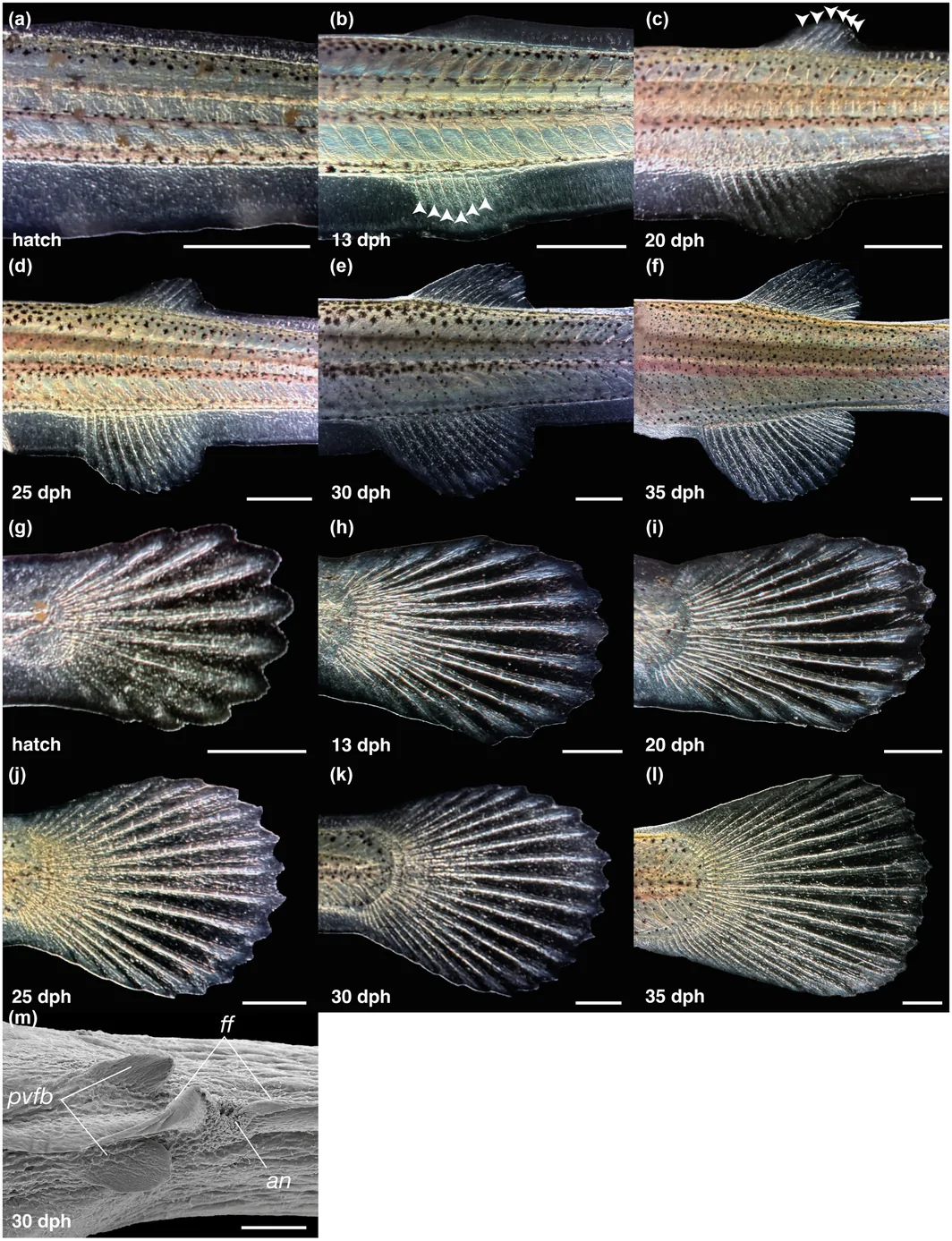
Fin development in *Tlaloc hildebrandi*. Sequential differentiation of the caudal, anal and dorsal fins from hatching to 35 days post‐hatch (dph). White arrowheads indicate fin ray primordia. an = anus; ff = finfold; pvfb = pelvic fin buds. Scale bars: (a–l) = 500 μm; m = 200 μm.

## 4 DISCUSSION

The embryonic development of *Tlaloc hildebrandi* from fertilisation to hatching lasted approximately 12 days under controlled laboratory conditions at 25°C, placing this species within the moderate‐to‐slow range of developmental rates reported for small teleosts (Byrne, 1978; Cunningham & Balon, 1985; Iwamatsu, 2004; Sezen & Olmez, 2012). Among non‐annual killifishes and other small freshwater fishes incubated at similar temperatures (24–26°C), the duration of embryogenesis typically ranges from approximately 5 to 16 days, depending on species and ecological context (Esmaeili et al., 2025). Rapidly developing species such as zebrafish (*Danio rerio*) complete development in as little as 3 days (Engeszer et al., 2007), while killifish, such as *Esmaeilius shirini*, hatch in about 5.5 days at 24°C (Esmaeili et al., 2025). Intermediate developmental periods are observed in species such as medaka (*Oryzias latipes*), *Cyprinodon variegatus* and *Fundulus xenicus*, which hatch within approximately 9–10 days under comparable temperatures (Iwamatsu, 2004; Koenig & Livingston, 1976; Lencer & McCune, 2018). The 12‐day incubation period observed in *T. hildebrandi* is more similar to that reported for other non‐annual killifishes, such as the mangrove killifish (*Kryptolebias marmoratus*) and the Arabian killifish (*Aphanius dispar*), both of which require roughly 12–14 days for embryogenesis (Alsakran et al., 2025; Mourabit et al., 2011), while some species, such as *Fundulopanchax gardneri*, may take up to 16 days at 25°C (Borisov et al., 2024).

The developmental rate observed in *T. hildebrandi* likely reflects its life‐history strategy and environmental conditions. Species of the genus *Tlaloc* are considered to exhibit an opportunistic reproductive strategy characterised by small body size, relatively early maturation and fractional spawning (Anzueto‐Calvo et al., 2024). Under such a strategy, a moderate embryonic period may facilitate steady recruitment without requiring specialised developmental arrests (Kapakos et al., 2024; Karageorgou et al., 2025). Unlike annual killifishes that inhabit ephemeral pools and exhibit diapause stages that can extend embryonic development for several weeks, *T. hildebrandi* occurs in permanent, spring‐fed streams and wetlands of the Chiapas highlands, where water bodies persist year‐round (Piller et al., 2024; Soria‐Barreto et al., 2023). Consequently, there is little selective pressure for diapause mechanisms, as in annual species such as *Austrofundulus limnaeus*, in which embryonic development may extend beyond 30 days (Podrabsky et al., 2017). Temperature likely also plays an important role in shaping developmental rates. Many small teleosts show strong thermal sensitivity during embryogenesis, and the approximately 12‐day development observed here may reflect adaptation to environmental conditions experienced by *T. hildebrandi* populations. Although *T. hildebrandi* is often associated with cooler, high‐altitude waters (14–19°C) in parts of its distribution (Velázquez‐Velázquez & Domínguez‐Cisneros, 2025), the occurrence of populations in warmer, lower‐altitude environments such as Ocosingo suggests that the species may tolerate a broader thermal range. Further studies across populations are needed to evaluate potential variation in developmental rates.

The egg diameter of *Tlaloc hildebrandi* (2.33 ± 0.03 mm) is comparatively large for small freshwater teleosts and non‐annual killifishes, suggesting a relatively high maternal investment per offspring. Among related or ecologically similar species, egg diameters commonly range from approximately 0.8 to 2.0 mm. For example, small eggs around 0.8–1.0 mm have been reported in species such as *Esmaeilius shirini*, while annual killifishes like *Nothobranchius furzeri* typically produce eggs measuring about 1.2–1.25 mm (Esmaeili et al., 2025; Vrtílek et al., 2017). Intermediate values around 1.4–1.7 mm occur in several species of *Aphanius* and other small cyprinodontiform fishes (Masoudi et al., 2018; Sanjarani Vahed et al., 2019; Sezen & Olmez, 2012). Larger eggs approaching 2 mm have been documented in species such as *Fundulus luciae*, *Fundulus xenica* and *Valencia robertae* (Byrne, 1978; Kapakos et al., 2024; Koenig & Livingston, 1976). In this context, the 2.33 mm eggs observed in *T. hildebrandi* fall at the upper extreme of the range reported for small teleost fishes.

Large egg size is generally associated with increased maternal provisioning and often results in larger larvae at hatching. This pattern is consistent with the relatively large hatchling size observed in *T. hildebrandi* (7.2 mm TL). Larger larvae may benefit from greater swimming capacity, improved predator avoidance and enhanced ability to locate food during early development (Brown et al., 2011; Karageorgou et al., 2025). Egg size has also been linked to environmental stability and temperature regimes. In several freshwater cyprinodontiforms, larger eggs are frequently associated with species inhabiting cooler or more stable aquatic environments, where slower embryonic development and extended incubation periods may occur (Kalogianni et al., 2010; Kapakos et al., 2024). The relatively large eggs of *T. hildebrandi* may therefore represent an adaptive strategy that enhances embryo resilience and larval performance in the cool, high‐altitude freshwater habitats characteristic of its natural distribution.

The eggs of *T. hildebrandi* possess adhesive chorionic filaments concentrated near the micropyle region (Figure 2a), a feature commonly observed in many freshwater teleosts that deposit demersal eggs. Adhesive eggs provide several ecological advantages, particularly in habitats with aquatic vegetation or moderate water movement (Bian et al., 2014; Huysentruyt & Adriaens, 2005). By attaching to submerged plants or debris, eggs remain in stable microhabitats and are less likely to be transported by currents into unfavourable environments (Berois et al., 2014; Esmaeili et al., 2025). This attachment also helps maintain eggs above fine sediments where oxygen availability may be reduced, thereby improving embryo survival (Furness, 2016). The concentration of adhesive filaments around the micropyle region may also facilitate successful fertilisation and early development (Berois et al., 2014).

The reproductive traits observed in *T. hildebrandi* are consistent with strategies commonly reported for non‐annual killifishes and other small freshwater teleosts inhabiting permanent aquatic environments. Non‐annual killifishes typically exhibit iteroparity, producing multiple clutches of eggs across extended reproductive periods rather than concentrating reproductive effort into a single spawning event (Anzueto‐Calvo et al., 2024; Furness, 2016). Many species are fractional or batch spawners, releasing small numbers of eggs repeatedly over days or months. *Tlaloc* species exhibit this strategy, reaching sexual maturity at small sizes and producing eggs whose number is positively correlated with female size and weight (Anzueto‐Calvo et al., 2024). This pattern has been documented in several cyprinodontiform fishes, including species such as *Fundulus xenicus* and *Esmaeilius shirini*, where females deposit a limited number of eggs per spawning event but maintain reproductive activity throughout the breeding season (Esmaeili et al., 2025; Koenig & Livingston, 1976). The reproductive pattern observed in *T. hildebrandi*, in which females deposited approximately 12–15 eggs per day during the reproductive period, is therefore consistent with the typical fractional spawning strategy characteristic of non‐annual killifishes. Non‐annual species also generally inhabit permanent or relatively stable aquatic habitats, such as springs, rivers, or vegetated wetlands, where adults can survive for multiple seasons and reproduce repeatedly. In such environments, reproduction is often seasonally synchronised with favourable environmental conditions, including temperature and photoperiod (Enriquez‐Valencia et al., 2022). The seasonal reproductive activity observed in *T. hildebrandi*, with spawning occurring primarily when water temperatures exceeded approximately 18°C, fits this general pattern of environmentally cued reproduction.

In contrast, annual killifishes living in temporary pools show a significantly different life‐history strategy. These species usually have a brief adult lifespan limited to the wet season and depend on specialised embryonic adaptations to endure habitat desiccation. Their embryos undergo diapause, a series of developmental arrest stages that enable eggs to stay viable in dried sediments for long periods (Berois et al., 2012; Furness, 2016; Murphy & Collier, 1997; Parenti, 2002). This approach differs markedly from the direct, continuous embryonic development observed in non‐annual species such as *T. hildebrandi*. Instead of entering diapause, embryos of non‐annual killifishes typically develop directly from fertilisation to hatching. However, some species may exhibit short periods of delayed hatching (latency) at later developmental stages (Furness, 2016). The lack of diapause and the relatively moderate developmental duration observed in *T. hildebrandi* therefore match the typical reproductive and developmental patterns of non‐annual killifishes adapted to stable freshwater environments.

The hatchling size observed in *Tlaloc hildebrandi* (7.20 ± 0.04 mm TL) is comparatively large relative to most small freshwater teleosts and killifishes. In many species of similar body size, larvae typically hatch at lengths ranging between approximately 3.0 and 6.5 mm. For example, larvae of *Cyprinodon variegatus* hatch at around 3.5–4.5 mm TL (Schnitzler et al., 2017), while *Fundulopanchax gardneri* and several *Aphanius* species hatch between roughly 3.0 and 6.0 mm TL (Alsakran et al., 2025; Esmaeili et al., 2025). Larger hatchlings approaching 6–6.5 mm have been reported in species such as *Valencia robertae* and *Fundulus grandis* (Brown et al., 2011; Kapakos et al., 2024). The hatchling size recorded for *T. hildebrandi*, therefore, places it among the largest larvae reported for small cyprinodontiform fishes, comparable only to some populations of the *Fundulus heteroclitus* (Marteinsdottir & Able, 1992). This relatively large size at hatching is consistent with the substantial egg diameter observed in this species (2.33 ± 0.03 mm), reflecting a high degree of maternal investment per offspring. Large hatchling size may provide several ecological advantages during early development. Larger larvae typically possess greater swimming capacity and improved manoeuvrability, which may enhance their ability to locate prey and avoid predators shortly after hatching (Huss et al., 2007). In addition, the relatively advanced development of sensory structures, fins and feeding apparatus at hatching in *T. hildebrandi* suggests that larvae are capable of initiating exogenous feeding rapidly after emergence (Krebs & Turingan, 2003). This pattern aligns with the observation that feeding behaviour began within the first hour after hatching, despite the continued presence of yolk reserves.

The duration of yolk‐sac absorption in teleost larvae varies widely among species and is influenced by egg size, developmental rate and environmental conditions. In many small teleost fishes, yolk reserves are depleted within one to several days after hatching. For example, larvae of the sheepshead minnow (*Cyprinodon variegatus*) may complete yolk absorption within approximately 24 h (Lencer & McCune, 2018), while in species such as the mangrove killifish (*Kryptolebias marmoratus*), yolk reserves are substantially reduced within 48–72 h post‐hatch (Mourabit et al., 2011). Similarly, in *Valencia robertae*, a non‐annual killifish with relatively large eggs, exogenous feeding begins within 24–48 h after hatching (Kapakos et al., 2024). In contrast, yolk reserves in *T. hildebrandi* were fully absorbed only after approximately 5 days, placing this species toward the longer end of the yolk utilisation period among comparable fishes. This extended period of yolk utilisation likely reflects the large initial egg size and the substantial energy reserves provided to the embryo. Larger yolk stores can function as a nutritional buffer during early larval development, allowing larvae to survive even if prey availability is initially low (Kamler, 2008; Martin, 1999; Yúfera & Darias, 2007). In *T. hildebrandi*, the presence of both residual yolk and early feeding activity indicates a mixed‐feeding phase in which larvae simultaneously utilise endogenous reserves while beginning exogenous feeding. Such a strategy may enhance early survival by reducing the risk of starvation during the critical transition from embryonic to independent feeding (Yúfera & Darias, 2007). In stable freshwater habitats, this combination of relatively large hatchling size and prolonged mixed‐feeding period may provide an advantage by allowing larvae to gradually transition to active foraging while still relying on maternal energy reserves. The ability of *T. hildebrandi* larvae to initiate exogenous feeding within the first hour after hatching represents an important developmental trait that may confer several ecological advantages during early ontogeny.

The sequence of fin differentiation observed in *T. hildebrandi*, in which the anal fin develops first, followed by the dorsal fin and finally the pelvic fins, is consistent with developmental patterns reported for other killifishes and many cyprinodontiform fishes. In several non‐annual species, including *Valencia robertae*, the anal fin is typically the first median fin to show ray primordia, while the dorsal fin differentiates shortly afterwards (Kapakos et al., 2024). Pelvic fins generally appear later during the larval period and are often the last paired fins to develop (Esmaeili et al., 2025). Similar sequences have been documented in other small teleosts such as *Cyprinodon variegatus* and *Fundulopanchax gardneri*, where dorsal and anal fins differentiate during post‐hatching development while pelvic fins emerge later as larvae approach the juvenile stage (Borisov et al., 2024; Lencer & McCune, 2018; Schnitzler et al., 2017). The delayed appearance of pelvic fins is considered a common feature of cyprinodontiform ontogeny (Esmaeili et al., 2025; Kapakos et al., 2024). Median fins, such as the anal and dorsal fins, contribute earlier to stabilisation and propulsion during swimming, whereas pelvic fins play a greater role in manoeuvrability and fine‐scale locomotion that becomes more important as juveniles grow and adopt more active swimming behaviours (Kapakos et al., 2024). The developmental sequence documented for *T. hildebrandi*, therefore, appears to follow a conserved ontogenetic pattern typical of killifishes, further supporting the interpretation that the species exhibits a conventional teleost developmental trajectory despite the absence of previous detailed descriptions for the family Profundulidae.

The reproductive characteristics observed in *T. hildebrandi*, including fractional spawning, relatively small daily egg batches, adhesive demersal eggs and the absence of parental care, are consistent with strategies commonly associated with fishes inhabiting freshwater systems or high‐altitude streams (Koenig & Livingston, 1976). Adhesive eggs are particularly advantageous in environments where moderate water flow or dense vegetation occurs, as chorionic filaments allow eggs to anchor securely to substrates such as aquatic plants or debris. This attachment helps prevent displacement by currents and maintains embryos within favourable microhabitats where oxygen availability and shelter from predators may be greater (Rizzo et al., 2002; Bian et al., 2014; Esmaeili et al., 2025). Fractional spawning, in which females release small batches of eggs repeatedly throughout the reproductive season, can function as a risk‐spreading strategy in environments where conditions may fluctuate (Anzueto‐Calvo et al., 2024). By distributing reproductive effort across multiple spawning events, the likelihood increases that at least some offspring encounter optimal conditions for survival. This strategy is often associated with relatively large eggs and high maternal investment per embryo, resulting in well‐developed larvae at hatching (Marteinsdottir & Able, 1992).

Detailed descriptions of embryonic and larval development, such as the one offered in this study, provide essential biological information that supports the conservation and management of endemic or endangered freshwater fishes. Early developmental stages often represent the most vulnerable phase of the life cycle, and understanding their morphological progression and environmental requirements is critical for both in situ and ex situ conservation efforts. Developmental staging series allow researchers to determine key parameters such as incubation duration, temperature sensitivity, oxygen requirements and the timing of important developmental transitions. This information is particularly valuable for establishing captive breeding protocols, creating ex situ safety populations and coordinating larval rearing conditions to maximise survival during artificial propagation programs.

For *Tlaloc hildebrandi*, several aspects of early development identified in this study have direct applications for conservation practice. The relatively long embryonic period (12 days at 25°C) and large egg size indicate a substantial dependence on stable environmental conditions during incubation, suggesting that habitat protection efforts should prioritise the maintenance of water quality and thermal stability in spawning sites. The presence of adhesive eggs attached to submerged vegetation highlights the importance of preserving microhabitats that function as natural spawning substrates. In addition, the observed mixed‐feeding phase and the delayed yolk‐sac absorption (5 dph) define a critical window during which larvae are particularly sensitive to food availability and environmental stress, providing a target period for optimising feeding regimes in captive propagation and for evaluating recruitment success in the wild. Finally, the early functionality of the feeding apparatus and rapid initiation of exogenous feeding suggest that larvae may be suitable for early‐stage rearing and potential reinforcement programs, provided that appropriate live prey and water conditions are maintained. These findings offer practical guidelines for both habitat management and the development of ex situ conservation strategies for this endangered species.

## 5 CONCLUSION

This study provides the first comprehensive description of embryonic and larval development in a species of profundulid fishes, establishing a detailed morphological and temporal framework from fertilisation to the juvenile stage for the Chiapas Killifish, *Tlaloc hildebrandi*. Development proceeded through a direct, non‐diapause trajectory over approximately 12 days at 25°C, followed by a rapid transition to functional independence during the larval period. The combination of large egg size, relatively large hatchling size, prolonged yolk utilisation and early onset of exogenous feeding indicates a life‐history strategy characterised by high maternal investment and enhanced early survival capacity. Morphological development, including head specialisation, sensory system differentiation and fin formation, followed patterns consistent with other non‐annual cyprinodontiform fishes. These developmental traits are closely aligned with the ecological context of *T. hildebrandi*, a species inhabiting stable, high‐altitude freshwater systems. The absence of diapause, combined with fractional spawning and adhesive demersal eggs, supports an opportunistic reproductive strategy adapted to permanent habitats with seasonal environmental cues. The relatively advanced condition of larvae at hatching, together with a mixed‐feeding strategy, likely enhances recruitment success under conditions where early survival is critical. Beyond its biological significance, this work provides essential baseline information for the conservation of *T. hildebrandi*, an endemic and threatened species. More broadly, these findings contribute to the understanding of life‐history evolution in non‐annual killifishes and highlight the importance of early developmental studies for the effective management and conservation of freshwater biodiversity.

## AUTHOR CONTRIBUTIONS

R.A.C.T.: Conceptualisation, Formal Analysis, Investigation, Methodology, Writing – Original Draft, Writing – Review & Editing, Visualisation. G.G.M.: Conceptualisation, Methodology, Resources, Writing – review & editing. M.E.C.P.: Conceptualisation, Methodology, Resources, Supervision, Validation, Writing – Original Draft, Writing – review & editing. All authors read and approved the final manuscript. M.I.B.D.M.: Writing – review & editing, Visualisation. J.A.: Investigation, Methodology, Writing – Original Draft, Writing – Review & Editing. A.A.G.D.: Resources, Writing – Review & Editing. W.A.M.: Conceptualisation, Formal Analysis, Investigation, Methodology, Supervision, Writing – Review & Editing.

## FUNDING INFORMATION

The authors declare that no funds, grants or other support were received during the preparation of this manuscript.

## CONFLICT OF INTEREST STATEMENT

The authors declare no conflict of interest.

## Data Availability

The data that support the findings of this study are available from the authors upon reasonable request.
